# Two-Layer Edge Intelligence for Task Offloading and Computing Capacity Allocation with UAV Assistance in Vehicular Networks

**DOI:** 10.3390/s24061863

**Published:** 2024-03-14

**Authors:** Xiaodan Bi, Lian Zhao

**Affiliations:** Department of Electrical, Computer and Biomedical Engineering, Toronto Metropolitan University, Toronto, ON M5B 2K3, Canada; xiaodan.bi@torontomu.ca

**Keywords:** mobile-edge computing (MEC), task offloading, resource allocation, duelling deep Q-learning

## Abstract

With the exponential growth of wireless devices and the demand for real-time processing, traditional server architectures face challenges in meeting the ever-increasing computational requirements. This paper proposes a collaborative edge computing framework to offload and process tasks efficiently in such environments. By equipping a moving unmanned aerial vehicle (UAV) as the mobile edge computing (MEC) server, the proposed architecture aims to release the burden on roadside units (RSUs) servers. Specifically, we propose a two-layer edge intelligence scheme to allocate network computing resources. The first layer intelligently offloads and allocates tasks generated by wireless devices in the vehicular system, and the second layer utilizes the partially observable stochastic game (POSG), solved by duelling deep Q-learning, to allocate the computing resources of each processing node (PN) to different tasks. Meanwhile, we propose a weighted position optimization algorithm for the UAV movement in the system to facilitate task offloading and task processing. Simulation results demonstrate the improved performance by applying the proposed scheme.

## 1. Introduction

The Internet of Things (IoT) [1] has been a promising research area in recent years. People, data, and devices can all connect to the Internet and one another in an IoT system. According to Cisco’s Annual Internet Report, global IoT connections are expected to reach 14.7 billion by 2023, with an average of 1.8 IoT connections per person [2]. Driving or walking on the road is one of the most active scenarios where people use smartphones and tablets for some transportation needs. Hence, the development of IoT also promotes intelligent transportation systems (ITSs) [3]. The exponential growth of users and devices on the ground has led to a surge in the demand for real-time processing and low-latency services. To meet these evolving requirements, traditional server architectures face significant challenges in terms of scalability, latency, and resource utilization. Mobile edge computing (MEC) [4] has emerged as a promising solution to address the limitations of traditional server architectures [5]. By deploying edge computing resources close to users, MEC can achieve the offloading and processing tasks in a distributed manner to reduce latency, enhance response, and enable high-bandwidth delivery services.

Despite its numerous benefits, MEC also faces certain limitations. One of the primary challenges is ensuring efficient task offloading and load balancing in a dynamic and heterogeneous environment. Allocating tasks to the most appropriate edge resources while considering factors such as network conditions, device capabilities, and energy constraints requires task-offloading decision-making mechanisms. Additionally, it is equally important to consider the computing resource allocation on each processing node (PN). Efficient computing resource allocation plays a vital role in improving system performance, enhancing user experience, and achieving overall optimization in MEC environments. Several studies have focused on offloading decisions or resource allocation, respectively, demonstrating progress in these two fields. Furthermore, solutions that jointly address task offloading decisions and resource allocation have emerged. Although most of them adopt a two-layer strategy, various optimization algorithms are widely utilized, or one of the layers uses RL algorithms to improve adaptability and efficiency. This paper proposes an RL-based two-layer collaborative task offloading and computing resource allocation optimization strategy. It not only solves two major issues by RL methods, respectively, but also combines a moving UAV to assist the system scenario and, finally, uses the proposed two-layer edge intelligence scheme to obtain task offloading decisions and resource allocation decisions. The major contributions of this paper are as follows:

(1) We propose a two-layer edge intelligence scheme to perform the offloading decision and computing capacity allocation. The proposed scheme reduces the service delay of tasks and increases the average utility of the system. The first layer makes offloading decisions for the newly generated tasks. The second layer reallocates the computing capacity intelligently to the unfinished tasks and the newly arrived tasks.
(2) We propose a weighted position optimization algorithm for the UAV movement based on the computing capacity and the remaining data of each PN. The UAV will move closer to the preferred location that needs more computing capacity, which can make the UAV perform task offloading and task processing more efficiently.
(3) This work fully considers various scenarios, taking into account both vehicle and pedestrian wireless device users, and deploying multiple RSUs and a UAV to collaborate within the scenario.

The rest of the paper is organized as follows: Section 2 introduces some related work. Section 3 describes the system model. Section 4 formulates the target problem of maximum the total utility of the system. Section 5 presents the collaborative task processing strategy. Section 6 further proposes to use the duelling DQN to solve the problem of computing capacity allocation. Section 7 shows the simulation results, and Section 8 concludes the paper.

## 2. Related Work

In a dynamic and heterogeneous environment, task offloading needs to consider how to allocate computing tasks between mobile devices and MEC servers dynamically and adaptably. Some works have proposed solutions with various algorithms. The policy gradient algorithm, as one of the reinforcement learning (RL) algorithms, was used to obtain a high-quality task offloading strategy in [6]. In [7], the authors proposed an RL-based intelligent online offloading scheme that can make optimal offloading actions in the MEC system powered wirelessly. In [8], an enhanced dynamic niche-based self-organizing learning algorithm was introduced to speed up the search for the optimal task offloading policy. In [9], the proposed approach dynamically adjusted the offloading decisions for all tasks according to the data parameters of the current task. In both [8,9], the Lyapunov optimization algorithm was used to ensure queue stability. In [10], Pham et al. developed a low-complexity distributed offloading strategy using a game-theoretic approach. The offloading decision was described as an exact potential game problem while the optimal offloading ratio and resource allocation were determined by a subgradient method. In [11], Yu et al. proposed a task scheduling and offloading scheme which can find the best sub-task offloading sequence and the suitable service nodes. In [12], the authors proposed a concept of dependence that takes into account the correlation between tasks. By employing graph convolutional neural networks to enhance the deep reinforcement learning (DRL) model, the application structure and the dependence features between tasks can be captured.

In addition, computing resource allocation is an important aspect of MEC that is also considered in this paper. There are many studies discussing how to improve this point. Sharif et al. proposed a scheme that adaptively allocates available resources by prioritizing incoming requests [13]. In [14], Chen et al. established a two-stage bargaining-based incentive scheme for task offloading and cooperative computing. Both user devices and local MEC servers attempt to maximize their utilities in the first stage. When the tasks on the local MEC server are overloaded, it will enter the second stage and cooperate with other MEC servers to perform load balancing and maximize the utilities of all MEC servers. In [15], the authors presented a market model of MEC resources where the computing resources are suppliers while the users are buyers, and the microeconomic theory is used to obtain an effective budget allocation scheme for customers to maximize utility within a given budget. A resource allocation based on vehicular cloud computing was proposed in [16]. This work was optimized from both the provider’s and users’ perspectives, filling the gaps in the previous research. In [17], the authors proposed a resource allocation technique for SDN-enabled fog computing with collaborative machine learning, integrated with the SDN-enabled fog computing environment. For cloud/edge computing resource management, a Stackelberg game was formulated with cloud/edge computing service providers as the leaders and users as followers in [18]. In [19], Wang et al. introduced a DRL-based resource allocation strategy that adaptively allocates computing and network resources, reduces average service time, and balances resource utilization in diverse MEC environments. In [20], Fang et al. treated the objective issue of maximizing the aggregate offloading benefits as a multiuser computing task offloading game. It was demonstrated to be an exact potential game with at least one pure-strategy Nash equilibrium (NE) solution.

The above papers discuss various methods to optimize the task offloading and computing resource allocation problems. There are also some papers discussing the offloading scheduling and resource allocation together. The issue of resource allocation and task offloading in an aerial-based MEC system was studied in [21]. The objective was to reduce the energy consumption of the ground devices as much as possible while maintaining performance and meeting offloading resource limits. The scenario of this work is suitable for remote areas. The dual Uu/PC5 interface offloading and resource allocation strategy in the vehicle edge computing system was proposed in [22]. They derived the closed-form expressions of transmission power of the Uu interface, packet transmit frequency of the PC5 interface, and CPU computation frequency in the resource allocation. Also, the offloading ratio matrix was obtained by utilizing the PC5 interface-based greedy offloading algorithm. This work focused on vehicles exchanging data and controlling information with the RSU using the Uu interface and each other through the PC5 interfaces. In [23], the authors utilized a stochastic mixed-integer nonlinear programming problem to simultaneously optimize radio resource allocation, elastic computation resource scheduling, and task offloading decisions. The original problem was broken down into four individual subproblems using the Lyapunov optimization theory and solved by matching games and convex decomposition techniques. In [24], Gao et al. proposed a two-layer optimization algorithm to solve this problem by minimizing the task completion delay and energy consumption. In [25], the authors looked into the joint issues of computation offloading, cache selection and transmission power, and CPU frequency allocation for cloud-edge heterogeneous network computing systems. To reduce the processing latency and energy consumption, a joint optimization technique based on sequential quadratic programming and the deep Q-network (DQN) was proposed together with a two-level alternation approach framework. A joint optimization problem was formulated by accounting for both system efficiency and fairness in [26]. They proposed a two-level algorithm: the upper-level algorithm searches preferable offloading schemes globally by evolutionary policies, while the lower-level algorithm generates resource allocation strategies that utilize server resources impartially.

## 3. System Model

**Figure 1:**
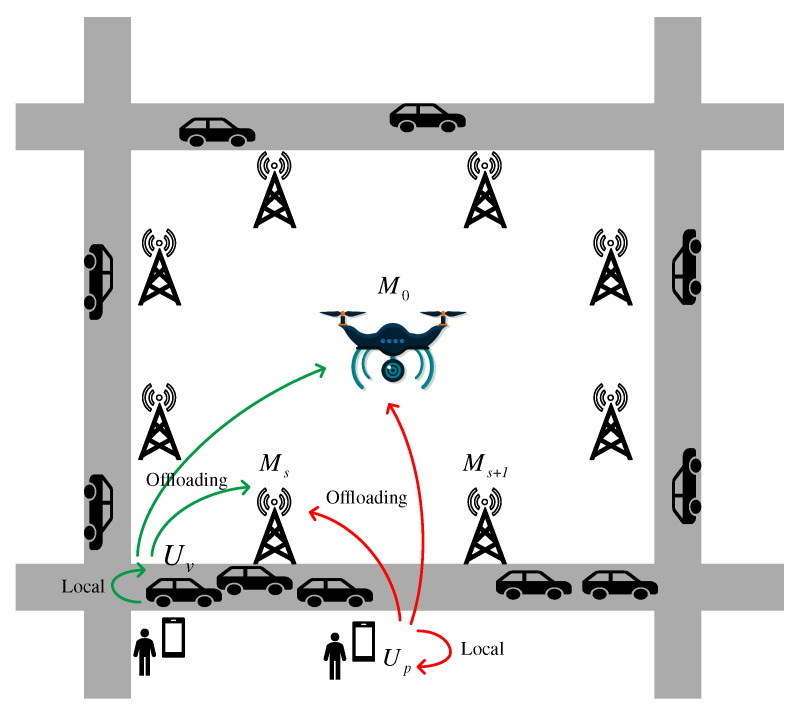
System model.

The air–ground network architecture is shown in Figure 1, which consists of a UAV in the air and RSUs deployed on the ground as the MEC servers to provide computing resources, and pedestrians and vehicles as users. UAV improves its service quality by moving in the air to cater to users’ computing demands. The RSUs, equipped with computing resources, provide seamless communication and computing service coverage for vehicles and pedestrians on the road. We can express all MEC servers, UAV and RSUs, as $M_{s}$, the index set S={0,1,…,s,…,S}. Hence, there are S+1 servers available in the system. The index s=0 refers to the UAV serving the user, and RSU otherwise. Let $U_{p}$ denote the pedestrian user (P-User), where $N_{p}=\left\{1,\ldots ,N_{p}\right\}$, and $U_{v}$ denotes the vehicle user (V-User), where $N_{v}=\left\{1,\ldots ,N_{v}\right\}$. Then, all users can be represented as $U_{u}$; the set is designated as $N_{u}$$=\{1,\ldots ,N_{p},N_{p}+1,\ldots ,N_{p}+N_{v}\}$, and let $N_{u}$$=N_{p}+N_{v}$. We adopt a discrete time slot model expressed as T={1,…,t,…T} and the duration of each time slot is defined as τ.

We can assume that P-Users, V-Users, RSUs, and the UAV all can process the tasks, denoted as PN. At the beginning of each time slot, all PNs will exchange basic information, such as the location and velocity, through hello beacon packets. The task generated by users can be processed locally or be offloaded to the MEC servers, either to the RSUs or UAV. In the model proposed in this paper, a computation service session for a task includes three steps: (1) Offloading: When a user generates a computation task, if it is decided to process locally, the user will process the task itself and there is no need to offload computation data; if it is decided to offload to the MEC servers for processing, the task computation data will be offloaded to the selected MEC server. (2) Queuing: After the computation task is fully offloaded, it needs to be queued in the selected PN until the PN makes the computing capacity allocation. (3) Computing: After the offloading time of each time slot, PNs will reallocate computing resources to unfinished tasks and newly offloaded tasks. Due to the small data size of the results compared to the computation tasks, we can ignore the delay in downloading the result. The important notations are defined in Table 1.

Since the P-Users, V-Users, RSUs, and UAV all have the ability to process the tasks, we need to make the offloading decision when the task is generated. There are four possible decisions:

1. P-Users: Since the devices of pedestrians have a weaker computing capacity than other PNs, the task can be processed locally or uploaded to MEC servers. We can assume that pedestrian devices can only process the tasks generated locally. Due to the high urgency of pedestrian demand, we can assume that the previous unfinished task can be discarded. Thus, there is no queuing time when the P-Users process the task locally.
2. V-Users: Although the computing capacity of the devices of V-Users is stronger than P-Users, it is still weaker than MEC servers. The tasks generated by vehicles can be processed locally or uploaded to MEC servers. And since the tasks on the vehicles are various, the unfinished tasks will keep processing. Hence, we need to consider the queuing time when the V-Users process the task locally.
3. RSU: When the RSU is selected as the PN, the task generated by the user needs to be offloaded to the RSU, and then it shares the computing capacity with unfinished tasks as the newly arrived task.
4. UAV: UAV has the strongest computing capacity compared to the RSUs. When it is selected as the PN, the task generated by the user also needs to be offloaded to the UAV and it shares the computing capacity with unfinished tasks as the newly arrived task. The UAV will perform small movements in the air to better complete the task.

## 4. Problem Formulation

Our objective is to offload and process tasks efficiently. Hence, the time of exchanging hello beacon packets including the mobility information and making decisions can be ignored in comparison. In this section, we will discuss the communication and cost model, define the utility function, and set it as the objective problem formulation.

### 4.1. Communication Model

The computation tasks will be generated at the beginning of each time slot. If the task is decided to offload to the MEC servers for processing, according to the Shannon capacity [27], the offloading data rate between the user $U_{u}$ and MEC Server $M_{s}$ can be expressed as

(1) $$R_{u}^{s}\left(t\right)=B_{s}\log_{2}\left(1+\frac{p_{u}\left(t\right)h_{u}^{s}\left(t\right)}{\sum_{u^{\prime }\neq u}p_{u^{\prime }}\left(t\right)h_{u^{\prime }}^{s}\left(t\right)+\sigma^{2}}\right),$$

where $B_{s}$ is the allocated communication bandwidth, $p_{u}$ represents the offloading transmit power of user $U_{u}$, $h_{u}^{s}$ is the uplink channel gain between the user $U_{u}$ and the MEC server $M_{s}$, and $\sigma^{2}$ is the Gaussian noise power.

Assuming that the location of the user $U_{u}$ is $L_{u}\left(t\right)$ and the location of the MEC server $M_{s}$ is $L_{s}\left(t\right)$, the distance between the user $U_{u}$ and MEC server $M_{s}$ is

(2) $$d_{u}^{s}=\left|\right|L_{u}\left(t\right)-L_{s}\left(t\right){\left|\right|}^{2},$$

where $\left|\right|\cdot {\left|\right|}^{2}$ is the Euclidean distance between two points.

1. UAV communication model: In this case, the uplink channel gain between the user $U_{u}$ and the UAV $M_{s},s=0$ can be expressed as [28]
  (3) $$h_{u}^{s}\left(t\right)=\frac{\phi_{s}}{d_{u}^{s}},\;\;s=0.$$
  where $\phi_{s}$ is the channel power gain at a reference distance of 1 metre.
2. RSU communication model: In this case, the propagation model follows from 3GPP standards [29]. The path loss in dB between the user and the RSU can be computed as
  (4) $$PL\left(d\right)=40\log_{10}d+21\log_{10}f_{c}+80$$
  where *d* is the distance in kilometres between the user and the RSU, and $f_{c}$ is the carrier frequency in MHz. The shadowing fading is ignored in this scenario. Then, the uplink channel gain between the user $U_{u}$ and the RSU $M_{s},s\in \left(1,S\right)$ can be expressed as
  (5) $$h_{u}^{s}\left(t\right)=10^{-PL(d_{u}^{s})/10},\;\;s\in \left(1,S\right).$$

### 4.2. Cost Model

In the proposed scheme, the service delay is composed of three parts: offloading delay, queuing delay, and processing delay.

**Figure 2:**
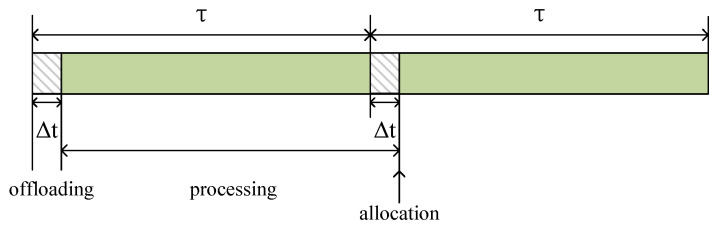
An example of the task offloading, processing, and computing capacity allocation.

1. Offloading delay
  Since all P-Users, V-Users, RSUs, and UAV can be PN, let $x_{u}^{q}\left(t\right)$ indicate offloading the task generated by user $U_{u}$ to the PN *q*, where $q\in \left\{U_{p},U_{v},M_{s}\right\}$. $x_{u}^{q}\left(t\right)=1$ indicates that PN *q* is selected to process the task generated by user $U_{u}$, or else $x_{u}^{q}\left(t\right)=0$. Let $W_{u}\left(t\right)$ denote the size of computation tasks generated at user $U_{u}$ at time slot *t*. With different offloading decisions, the offloading delay can be calculated as follows:
  (6) $$T_{u}^{o}\left(t\right)=\left\{\begin{array}{cc} 0, & q\in \left\{N_{p},N_{v}\right\}\\ \frac{W_{u}\left(t\right)}{R_{u}^{s}\left(t\right)}, & q\in \left\{M_{s}\right\}. \end{array}\right.$$
2. Queuing delay
  We choose the first small part of each time slot as the time to wait for offloading, denoted as Δt=μτ,μ∈(0,1). During Δt, the unfinished tasks from the previous time slot can still be processed, and the newly arrived tasks use this time offloading. After Δt, PN will allocate resources to unfinished tasks and newly arrived tasks. Figure 2 shows an example of the scenario of task offloading, processing, and reallocation of computing resources. If $T_{u}^{o}>\Delta t$, the task can be considered a processing failure.
3. Processing delay
  According to Figure 2, after Δt of each time slot, the PN will allocate the computing resources to the unfinished tasks and the newly arrived tasks. In the unfinished or newly arrived tasks, computing resources will be allocated proportionately to the task size. Let $w_{u}^{q}\left(t\right)\in \left\{0,1\right\}$ represent the ratio of computing resources allocated to the task generated by user $U_{u}$ on PN *q*. Assuming this task can be completed in the *K*th time slot, the processing delay of the task $W_{u}\left(t\right)$ is
  (7) $$T_{u}^{p}\left(t\right)=\left\{\begin{array}{cc} \frac{XW_{u}\left(t\right)}{w_{u}^{q}\left(t\right)C_{q}}, & K=1\\ \left(K-1\right)\tau +\frac{XW_{u}\left(t\right)-\tau C_{q}\sum_{k=2}^{K}w_{u}^{q}\left(t+K-2\right)}{w_{u}^{q}\left(t+K-1\right)C_{q}}, & K\geq 2 \end{array}\right.$$
  where $C_{q}$ denotes the computing capacity (CPU-cycle frequency) of PN *q* and X denotes the number of computing cycles needed to execute 1 bit of data.

Therefore, the total service delay to complete task *k* can be expressed as

(8) $$T_{u}\left(t\right)=\Delta t+T_{u}^{p}\left(t\right).$$

### 4.3. Utility Function Model

The utility of the proposed strategy can be expressed as the revenue received from executing the two-layer edge intelligence scheme minus the penalty, which can be expressed as follows:

(9) u(t)=r(t)−n(t)

where r(t) is the revenue from executing the two-layer edge intelligence scheme, which is defined as the delay difference between executing the proposed scheme successfully and the local processing model:

(10) $$r\left(t\right)=\sum_{u=1}^{N}\beta_{u}\left[T_{u}^{l}\left(t\right)-T_{u}\left(t\right)\right]$$

where $\beta_{u}$ is a variable. When it is equal to 1, the task is successfully processed, and 0 means failure. $T_{u}^{l}\left(t\right)$ is the service delay of local processing. The penalty term n(t) captures the consequences of task failure. We consider three cases as task failure in this paper; the vehicle leaves the modelled region, the task of P-Users is not completed locally within a time slot, or the task offloading time is larger than Δt. It can be written as

(11) $$n\left(t\right)=\sum_{u=1}^{N}\left(1-\beta_{u}\right)F_{u}\left(t\right)+\sum_{u=1}^{N}\beta_{u}\left(\Delta t-T_{u}^{o}\left(t\right)\right)$$

where the first term $F_{u}\left(t\right)$ is the failure penalty, where $F_{u}\left(t\right)=0$ when the task is completed successfully and $F_{u}\left(t\right)=\alpha T_{u}^{l}\left(t\right)$ if the task fails. α is a weight factor between 0 and 1. The second term is the sum of the difference between Δt and offloading time.

### 4.4. Problem Formulation

Currently, we can formulate the objective problem as maximizing the total utility of the system. There are two decisions, the task offloading decision and the ratio of computing capacity. The corresponding objective function can be formulated as follows:

(12a) $$\begin{array}{cc} \; & \max_{\left\{x_{u}^{q}\left(t\right),w_{u}^{q}\left(t\right)\right\}}u\left(t\right) \end{array}$$

(12b) $$\begin{array}{cc} \; & x_{u}^{q}\left(t\right)\in \left[0,1\right],\;q\in \left\{U_{p},U_{v},M_{s}\right\} \end{array}$$

(12c) $$\begin{array}{cc} \; & w_{u}^{q}\left(t\right)\in \left(0,1\right),\;q\in \left\{U_{p},U_{v},M_{s}\right\} \end{array}$$

(12d) $$\begin{array}{cc} \; & \sum_{q=1}^{N+S}x_{u}^{q}\left(t\right)=1,\;\forall U_{u} \end{array}$$

(12e) $$\begin{array}{cc} \; & \sum_{u=1}^{N}w_{u}^{q}\left(t\right)=1,\;\forall n \end{array}$$

(12f) $$\begin{array}{cc} \; & \left|\right|L_{u}\left(\tau +1\right)-L_{u}\left(\tau \right){\left|\right|}^{2}\leq V_{u}^{max}\tau ,\;\forall \tau \end{array}$$

Constraint Equation (12b) represents the offloading decisions, where $x_{u}^{q}\left(t\right)=1$ means that PN *q* is selected or else $x_{u}^{q}\left(t\right)=0$. Equation (12c) represents the ratio of computing resources that will be allocated to task *k* at time slot *t*. Equation (12d) constrains that only one PN will be selected, and Equation (12e) is the constraint that the sum of computing resources allocated to each task at time slot *t* on PN *n* should be 1. Equation (12f) states that the UAV trajectory is limited by the maximum speed $V_{u}^{max}$. Since we have two decision vectors in the target problem formulation, the $x_{u}^{q}\left(t\right)$ and $w_{u}^{q}\left(t\right)$, determine the offloading decisions and the ratio of computing capacity, respectively, and we can divide the target problem into two layers to maximize the utility of the system model.

## 5. Collaborative Task Processing Algorithm

This section introduces the proposed RL-based collaborative task offloading and computing resource allocation optimization strategy. This strategy not only considers two major issues at the same time, but also explores the UAV trajectory optimization.

### 5.1. UAV Trajectory

The UAV can make small movements in the selected area to efficiently complete the computing tasks. Through its mobility, it can provide a more flexible and adaptable service to the changing network conditions than the fixed RSUs. Therefore, our design requirement is to find a preferred trajectory for the UAV to move to areas that need more computing capacity to alleviate the task processing pressure on the RSUs and the users. We can have all users and RSUs as a point set $L_{n}\left(t\right)=\left\{L_{u}\left(t\right),L_{s}\left(t\right)\right\},s\neq 0$, and the location of the UAV is $L_{0}\left(t\right)$. The total number of points in the set is Q=N+S. Due to the different computing power and available time of each PN, the movement of the UAV will also be affected by these factors. Hence, we can have weighted coordinates of users and RSUs and then calculate the weighted mean centre as the optimal location of the UAV. We can first denote the weight vector as $b_{n}\left(t\right)=\left\{b_{u}\left(t\right),b_{s}\left(t\right)\right\},s\neq 0$. Since we consider two factors in this paper, computing power and remaining data size at the beginning of each time slot, we can denote them as $b_{n1}$ and $b_{n2}$, respectively. According to the computing power, we can have the normalized computing power weight:

(13) $$b_{n1}\left(t\right)=1-\frac{C_{q}}{C_{s=0}},\;\;q\in \left\{U_{u},M_{s}\right\},s\neq 0.$$

Then, we can first calculate the size of computation data that can be processed in a time slot. When the V-User or RSU is the PN, the maximum data size that can be processed in a time slot by PN *q* is

(14) $$M_{q}\left(t\right)=\frac{\tau C_{q}}{X},\;\;q\in \left\{U_{v},M_{s}\right\},s\neq 0.$$

Then, the remaining data size on the V-User or RSU at the beginning of the next time slot can be expressed by

(15) $$D_{q}\left(t+1\right)=\max\left\{D_{q}\left(t\right)+\sum_{u=1}^{N}W_{u}^{q}\left(t\right)-M_{q}\left(t\right),0\right\},\;\forall q\in \left\{U_{v},M_{s}\right\},s\neq 0.$$

The remaining data size can be used to indicate how busy the PN is, so we can express the weight $b_{n2}$ according to the dynamic computing resource state:

(16) $$b_{n2}\left(t\right)=\left\{\begin{array}{cc} 0, & q\in U_{p}\\ \frac{D_{q}\left(t\right)}{M_{q}}, & q\in \left\{U_{v},M_{s}\right\},s\neq 0. \end{array}\right.$$

Since $b_{n1}$ and $b_{n2}$ are both normalized weight factors, we can have the weight vector $b_{n}\left(t\right)=b_{n1}\left(t\right)+b_{n2}\left(t\right)$. Then, the weighted mean centre of all the points, which is considered as the preferred location of the UAV, can be written as

(17) $$L_{0}\left(t\right)=\frac{\sum_{n=1}^{Q}b_{n}\left(t\right)L_{n}\left(t\right)}{\sum_{n=1}^{Q}b_{n}\left(t\right)}.$$

### 5.2. Offloading Decision

Since we have two decision vectors in the objective function and their goals are the same, we propose a two-step DQN model to solve the problem. First, it is possible to rephrase the task offloading decision issue as a Markov decision problem (MDP), assuming that the P-User and V-User that generated the tasks can make the task offloading decision in this scheme. MDP is generally defined in a tuple with four elements: $\left\{S,A,P,andR\right\}$. In this tuple, S is state space, A is action space, $P=\left\{p(s(t+1)|s(t),a(t))\right\}$ is the transition probabilities, and R is the reward function [30]. In the scenario of this work, the state space and the action space for offloading decisions can be characterized as follows.

(1) State space: In the subproblem of offloading decisions, the network state is composed of the newly generated tasks; the computing capacity of each PN and the set of PNs can be selected:
  (18) $$S\left(t\right)=\left\{G_{u},C_{q},PN_{u}\right\},$$
  where $G_{u}$ is the newly generated task and $PN_{u}$ is the set of PNs that can be selected by user $U_{u}$.
(2) Action space: The first action is the offloading decision $x_{u}^{q}\left(t\right)$ to the task. The action space can be expressed as $A\left(t\right)=\left\{a\left(t\right)|a\left(t\right)=x_{u}^{q}\left(t\right)\right\}$.

Therefore, DQN can make an action on the offloading decision based on the current environment state, and then update the environment state to $S’\left(t\right)=\left\{G_{u}^{\prime },C_{q},PN_{u}^{\prime }\right\}$, where $G_{u}^{\prime }$ is the newly arrived tasks and $PN_{u}^{\prime }$ is the updated set of PNs that can be selected. Given the wide state-action space and unknown transition probability of this MDP problem, DRL is required to determine the offloading decision policy π. Policy π is a mapping from state space to action space. Further, DQN, the improved Q-learning algorithm, is utilized here to find the offloading decision policy in this subproblem. DQN is the neural network to estimate a state-value function in a Q-learning framework. DQN is the process of finding the optimal policy approaching the maximum Q-value, and our objective is to find the offloading decision to obtain the maximum total utility. Hence, we can designate the reward function as

(19) R(t)=u(t).

The discounted return is

(20) $$U\left(t\right)=\sum_{t=0}^{\infty }\gamma^{k}R\left(t\right),$$

where γ∈(0,1). Therefore, the action-value function corresponding to policy π can be expressed as

(21) $$Q_{\pi }\left(s,a\right)=E_{\pi }\left[U\left(k\right)|S\left(t\right)=s,A\left(t\right)=a\right].$$

When the optimal Q value for the next time slot is known, the estimation value $Q^{*}$ can be found by applying the Bellman optimal equation for the action-value function.

(22) $$Q^{*}\left(s,a\right)=\max_{\pi }Q_{\pi }\left(s,a\right).$$

Then, the optimal policy is the one that can maximizes the Q value:

(23) $$\pi^{*}=\underset{\pi }{\arg\;\max}Q^{*}\left(s,a\right).$$

The neural networks of DQN are trained to enable $Q\left(s,a,\theta \right)\approx R+\gamma \max_{a}Q\left(s^{\prime },a;\theta^{\prime }\right)$[31]. The neural network weight θ is trained to minimize loss at the end of each learning iteration. The loss function can be formulated as follows:

(24) $$LF\left(\theta_{i}\right)=E\left[{\left(y_{i}-Q\left(s,a;\theta_{i}\right)\right)}^{2}\right],$$

where $y_{i}$ is the target action-value calculated by the network weight $\theta_{i-1}$, which decided from the previous iterations. Q(s,a;θ) is the evaluation Q-value of the neural network. The loss function’s gradient descent can be expressed as

(25) $$\nabla_{\theta_{i}}LF\left(\theta_{i}\right)=E\left[y_{i}-Q\left(s,a;\theta_{i}\right)\right)\nabla_{\theta_{i}}Q\left(s,a;\theta_{i}\right)].$$

Algorithm 1 shows the DQN algorithm’s procedure [30]. We take the newly generated task, and the computing capacity of each PN and the set of PNs can be selected as the neural network state space, and then we utilize the DQN to make the task offloading decision.
**Algorithm 1** DQN algorithm.  1:Initialize replay memory pool M  2:Initialize neural network weight θ and target weight $\theta^{\prime }=\theta$  3:**for** episode *i* = 1, *M* **do**  4:      **for** t=1,T **do**  5:            Obtain state $s_{t}$ from the environment  6:            Randomly select an action $a_{t}$ or determine $a_{t}=\arg\max_{a}Q\left(s_{t},a_{t};\theta \right)$  7:            Observe the reward $R_{t}$ with the action $a_{t}$ and obtain the next state $s_{t+1}$  8:            Store transition $\left(s_{t},a_{t},R_{t},s_{t+1}\right)$ in memory M  9:            Randomly sample a mini-batch $\tilde{M}$ of M10:            Update the evaluation network and perform $\nabla_{\theta_{i}}LF\left(\theta_{i}\right)$11:            Update the target network after *C* steps12:      **end for**13:**end for**14:**Output**: offloading decision strategy $a_{t}$.

### 5.3. Computing Capacity Allocation

Since there are unfinished tasks and newly arrived tasks on each PN, we need to allocate computing resources to complete all tasks. We can assume that PN is an individual who wants to maximize their utility and only has a full observation of its own states. Hence, the computing resources allocation process can be formulated as a partially observable stochastic game (POSG). Each PN’s utility at any given time slot τ depends on its own current state and the actions of other PNs in the scenario. The game will enter an entirely new stochastic state in the subsequent time slot, impacted by the selected actions of all PNs and the previous states. Thus, we design the second subproblem as a POSG which can be defined in a tuple with six elements <S, A, P, U, O, O> [32]. In this tuple,

1. State space: S is the set of possible states. For the computing capacity allocation issue at time *t*, the state space is the set of all PNs, $S\left(t\right)=\{S_{1}\left(t\right),\ldots ,S_{q}\left(t\right),\ldots ,S_{Q+1}\left(t\right)\}$. For each PN, its state is composed of the following three parts:
  (26) $$S_{q}\left(t\right)=\left\{s_{q}\left(t\right)=\left(J_{q}\left(t\right),D_{q}\left(t\right),\Gamma_{q}\left(t\right)\right)\right\}$$
  where $J_{q}\left(t\right)$ represents the newly generated tasks assigned to PN *q*, $D_{q}\left(t\right)$ represents the remaining data size of PN *q*, and $\Gamma_{q}\left(t\right)$ represents the previous computing resource allocation of PN *q*.
2. Action space: A is the finite set of joint actions. The global action space of all PNs A(t) can be defined as $A\left(t\right)=\{A_{1}\left(t\right),\ldots ,A_{q}\left(t\right)\ldots ,A_{Q+1}\left(t\right)\}$. For each PN, its action is the ratio of computing capacity:
  (27) $$A_{q}\left(t\right)=\left\{a_{q}\left(t\right)=w_{q}^{k}\left(t\right)\right\}.$$
  P is the transition probability function from the current state $S_{q}\left(t\right)$ to the subsequent state $S_{q}\left(t+1\right)$ after executing the action $A_{q}\left(t\right)$.
3. Observations: O is the finite set of observations. According to the proposed model, PN *n*’s observation is the information obtained from the assigned new tasks $J_{q}\left(t\right)$, the remaining data size $D_{q}\left(t\right)$, and the previous computing resource allocation state $\Gamma_{q}\left(t\right)$. Hence, it can be expressed as
  (28) $$O_{q}\left(t\right)≜\left\{J_{q}\left(t\right),D_{q}\left(t\right),\Gamma_{q}\left(t\right)\right\}.$$
  O∈[0,1] is the observation probability function. In this model, each PN can only observe its local information from $J_{q}\left(t\right),D_{q}\left(t\right),\Gamma_{q}\left(t\right)\;$, but not from other PNs.
4. Objective: Based on the state $S_{q}\left(t\right)$, PN seeks to maximize its utility by making optimal computing capacity allocation decisions. The PNs are non-cooperative due to partial observation and having no other PNs’ information. However, the target maximum utility of the overall system depends on not only the decision of one PN itself but also the decisions of other PNs. We denote $\pi =\{\pi_{1},\ldots ,\pi_{q},\ldots ,\pi_{Q+1}\}$ as the multi-policy of PNs, where $\pi_{q}$ denotes the allocation policy of PN *q*. Moreover, we use $\pi_{-q}^{*}=\left(\pi_{1}^{*},\ldots ,\pi_{q-1}^{*},\pi_{q+1}^{*},\ldots ,\pi_{Q+1}^{*}\right)$ to represent the allocation decisions of all other PNs except PN *q*. U is the immediate utility. Then, the maximum utility of PN *q* can be expressed as
  (29) $$\max_{\pi_{q}}U_{q}\left(\pi_{q},\pi_{-q}\right)=\lim_{T\to \infty }\frac{1}{T}E_{\pi_{q},\pi_{-n}}\left[\sum_{t=0}^{T}u_{q}\left(t\right)\right]$$
5. Local equilibrium of the POSG: When other PNs’ policies are determined, each PN independently makes its own computing capacity allocation policy to optimize the utility function. With the procedure of seeking all other PNs’ optimal policies, we can take the local equilibrium as the solution of the proposed POSG. The multi-policy $\pi^{*}=\left\{\pi_{1}^{*},\ldots ,\pi_{q}^{*},\ldots ,\pi_{Q+1}^{*}\right\}$ is a local equilibrium; then, for each PN *q*, the strategy $\pi_{q}^{*}$ such that
  (30) $$U_{q}\left(\pi_{q}^{*},\pi_{-q}^{*}\right)\geq U_{q}^{*}\left(\pi_{q},\pi_{-q}^{*}\right),\;\forall \pi$$
  This means that each PN’s action is the optimal choice for other PNs’ policies in local equilibrium. In other words, PN *q* cannot adopt any alternative policy $\pi_{q}^{\prime }$ to obtain a greater utility when other PNs’ policies maintain the same. It has been demonstrated that the local equilibrium of the POSG exists [33].

## 6. POSG for Computing Capacity Allocation

This section introduces the DRL-based algorithm that can solve the proposed optimal computing capacity allocation problem. Among various DRL algorithms for training agents, we choose DQN because the action space of the computing capacity allocation considered in this work is discrete, and DQN can stably train the neural network and converge to the optimal policy.

### 6.1. Preliminary

In the proposed POSG, each PN cannot observe the state of other PNs. DQN is an effective method for working in an uncertain stochastic environment. It can be seen from the previous section that each PN *q* has the objective to find the optimal policy $\pi_{q}^{*}:S\to A$ to maximize its utility. The value-state function is defined to quantify the long-term utility of PN *q*:

(31) $$V_{q}\left(s_{q},\pi_{q},\pi_{-q}\right)=E\left[\sum_{t=0}^{T}\gamma u_{q}\left(s_{q}\left(t\right),\pi_{q}\left(t\right),\pi_{-q}\left(t\right)\right)|s_{q}\left(0\right)=s_{q}\right]$$

where γ∈[0,1]. It can be seen from the previous section that the multi-policy $\pi^{*}$ can reach the local equilibrium. It also means that the state-value function can satisfy the following inequality with any policy $\pi_{q}$:

(32) $$V_{q}\left(s_{q},\pi_{q}^{*},\pi_{-q}^{*}\right)\geq V_{q}\left(s_{q},\pi_{q},\pi_{-q}^{*}\right),\;\forall s_{q}\in S_{q}$$

Since the DQN only trains the *Q*-values of observed state-action pairs rather than the actions that have not been taken, the learning speed is not enough. To accelerate the learning process, a duelling architecture is thus established. We can decompose Q(s,a) as the sum of the estimation of the state value function $V_{q}$ for each state *s* and the potential advantage function A(s,a) of each action *a* at a given state *s*.

(33) $$Q_{q}\left(s_{q},a_{q}\right)=V_{q}\left(s_{q}\right)+A\left(s_{q},a_{q}\right)$$

With duelling DQN, we can use these two streams to separate the estimator of these two elements. It is not required to determine the value of each action in this situation. This is especially useful for states whose actions do not appropriately impact the environment. However, we are unable to find V(s) and A(s,a) with a given Q(s,a) due to the issue of identification. To solve this problem, we can make the advantage function estimator have no advantage at the selected action and then subtract the average advantage of all other possible actions of this state:

(34) $$Q_{q}\left(s_{q},a_{q},\theta \right)=V_{q}\left(s_{q};\theta \right)+\left(A_{q}\left(s_{q},a_{q};\theta \right)-\frac{1}{|A_{q}|}\sum_{a_{q}^{\prime }\in A_{q}}A\left(s_{q},a_{q}^{\prime };\theta \right)\right)$$

where $|A_{q}|$ is the size of the action space. In this way, more reliable Q values for each action can be obtained by decoupling the estimation between two streams.

### 6.2. Dueling DQN for POSG

With the preliminary, we can use the duelling DQN architecture to solve the proposed POSG. Each PN aims to learn the optimal computing capacity allocation policy to maximize its own utility. The procedure of the proposed duelling DQN is described in Algorithm 2. Each learning episode starts with the network state initialized. In each step of an episode, each PN obtains the newly assigned tasks state $J_{q}\left(t\right)$, its local remaining data state $D_{q}\left(t\right)$, and previous computing capacity allocation state $\Gamma_{q}\left(t\right)$; and each PN obtains its current local observation $s_{q}\left(t\right)=\left\{J_{q}\left(t\right),D_{q}\left(t\right),\Gamma_{q}\left(t\right)\right\}$.

According to the state space, each PN obtains the action $a_{q}$ from the estimated *Q*-value $Q_{q}\left(s_{q},a_{q};\theta_{q}\right)$ with the ϵ-greedy policy. Executing the computing capacity allocation, each PN can obtain its current local utility $u_{q}\left(s_{q},a_{q}\right)$, the utility of all other PNs $u_{q^{\prime }}\left(s_{q^{\prime }},a_{q^{\prime }}\right),q\neq q^{\prime }$, and the subsequent local observation state $s_{q}^{\prime }$. Then, the current local state and action, together with the subsequent local observation state and the utility, are stored in the memory M as a tuple $<s_{q},a_{q},u_{q}\left(s_{q},a_{q}\right),s_{q}^{\prime }>$. The experience replay will randomly sample the in-memory data to generate mini-batches for updating network parameters θ. The utility of each step is accumulated into the total utility. Each PN first randomly selects an action, and then iteratively trains until it reaches the preset number of training steps and there are enough samples in its memory for at least one mini-batch. After training, each PN will learn to obtain great utility by seeking the optimal policy.
**Algorithm 2** Duelling DQN-based for the POSG.**Input:** replay memory pool M  1:Initialize neural network weight $\theta_{q}$ and $\theta_{q}^{-}$;  2:**for** episode ep=1,2,… **do**  3:      Obtain state $s_{q}\left(0\right)$ from the environment;  4:      **for** step t=1,…,T **do**  5:            **for** each PN *q* **do**  6:                  Obtain the new assigned tasks $J_{q}\left(t\right)$;  7:                  Obtain the previous computing capacity allocation state $\Gamma_{q}\left(t\right)$;  8:                  Obtain the current local observation $s_{q}\left(t\right)=\left\{J_{q},D_{q},\Gamma_{q}\right\}$;  9:                  Selects action $a_{q}$ with: $a_{n}=\epsilon -greedy\left(Q_{q}\left(s_{q},a_{q};\theta_{q}\right)\right)$;10:                  Execute the computing capacity allocation and obtain the local utility $u_{q}\left(s_{q},a_{q}\right)$;11:                  Obtain utility from other PNs $u_{q^{\prime }}=\left(s_{q^{\prime }},a_{q^{\prime }}\right),q^{\prime }\neq q$ and the subsequent local observation $o_{q}^{\prime }$;12:                  Stores transition tuple $<s_{q},a_{q},u\left(s,a\right),s_{q}^{\prime }>$ into M;13:                  Randomly sample a mini-batch $M_{n}$ from M;14:                  Update the network weights $\theta_{q}$ by performing gradient descent;15:                  Update target network parameters $\theta_{q}^{\prime }=\theta_{q}$ after *C* steps;16:            **end for**17:      **end for**18:**end for**19:**Output:** computing capacity allocation strategy $a=\{a_{1},\ldots ,a_{q}\ldots ,a_{Q+1}\}$.

## 7. Simulation Results

This section validates the effectiveness and efficiency of the proposed two-layer edge intelligence scheme for task offloading and processing. VISSIM simulates the vehicle traffic network, and a Python environment is built for the edge intelligence scheme simulation.

In the simulation, we consider a square area with four 1 km two-way lanes on all four sides, and eight RSUs uniformly distributed, as shown in Figure 1. In this paper, only the RSU near the user will be considered as the MEC server for offloading tasks, so the deployment of RSUs will only affect the transmission delay in terms of distance between the RSU and the user, and the deployment shape between multiple RSUs has no impact. The vehicle traffic network is created with the traffic volume of 400 vehicles and the velocities are distributed between 20 and 60 km/h uniformly. At the beginning of each time slot, the generation of tasks is modelled as a Poisson process, and the size of each task is randomly distributed between 100 and 500 KB. When the task fails, α can be taken as 0.1. Other parameters of system model settings are presented in Table 2.

The proposed strategy is trained in iterations by two layers. The learning process is set to be 1500 episodes and there are 100 steps in each episode. The utility of each step is accumulated into the total utility. The learning rate is 0.001 and the discount rate is 0.85. An adaptive ϵ-greedy algorithm is used to avoid falling into a suboptimal policy before the system has sufficient learning experience. ϵ is initially set to 1 and multiplied by 0.995 per time step until it reaches 0.1.

**Figure 3:**
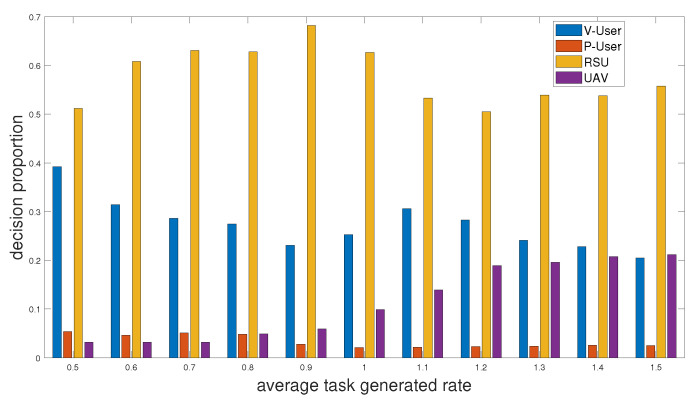
Distribution of offloading decision versus average task generated rate.

Figure 3 shows the distribution of offloading decisions made by the proposed scheme under different average task generated rates. The value of the average task generated rate ranges from 0.5 to 1.5, meaning the average of the number of tasks generated per user at the beginning of each time slot. We can see that as the average task generated rate increases, the offloading decision falls more and more on the UAV, but, overall, RSU is always the main choice for offloading tasks.

We can observe the effectiveness of the proposed two-layer edge intelligence scheme by comparing it with other schemes. In the following analysis, we use the four abbreviations “The proposed”, “NUM”, “OD”, and “Local” to refer to the four schemes:

(1) The Proposed: the proposed two-layer scheme in this work.
(2) NUM: No UAV movement in the scenario.
(3) OD: Only offloading decisions are executed. After offloading to the MEC server or deciding to process locally, if there are multiple pending tasks on the PN, they will be processed in sequence.
(4) Local: Only processing the tasks locally.

**Figure 4:**
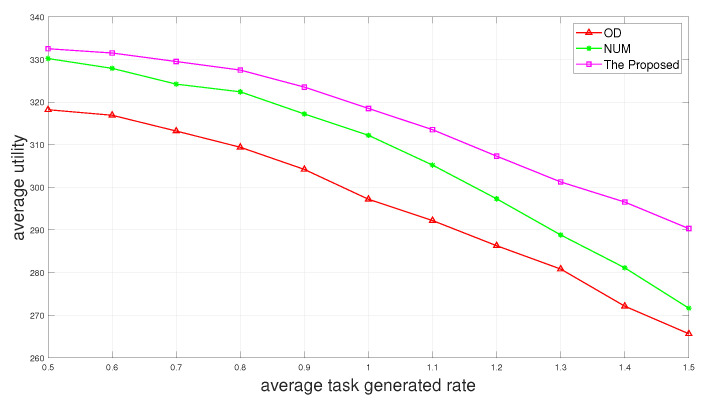
Average utility versus average task generated rate.

In Figure 4, we evaluate the performance of the proposed two-layer scheme “The Proposed” with “NUM” and “OD” as a function of average task generated rate. We can observe that the average utility decreases as the average task generated rate increases. It is because the larger the average task generated rate is, the more tasks are generated at time slot *t* and there are not enough resources in the system to complete all the tasks. This can also explain that when the average task generated rate is smaller, the average utility decreases to a smaller extent.

**Figure 5:**
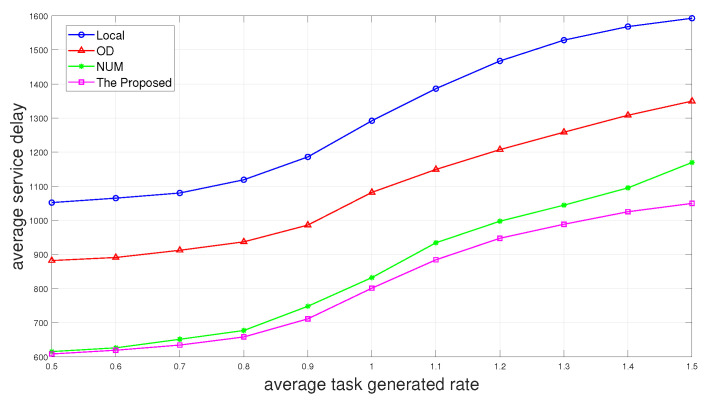
Average service delay versus average task generated rate.

Then, we add the scheme “Local”, which only processes the tasks locally to the comparison, and we can see in Figure 5 that the average service delay increases as the average task generated rate increases. The average service delay of “Local” is undoubtedly the largest, and the scheme of only performing the first layer of offloading decision “OD” is slightly smaller than it. The proposed two-layer scheme performs the best. The scheme “NUM” with no UAV movement performs almost the same as the proposed scheme when the average task generated rate is small. It is because there are fewer tasks generated at this time, and we can observe in Figure 3 that the offloading decisions fall less on the UAV when the average task generated rate is small. Therefore, when fewer tasks are generated, we can choose to not use UAV, or UAV does not move to reduce energy consumption.

**Figure 6:**
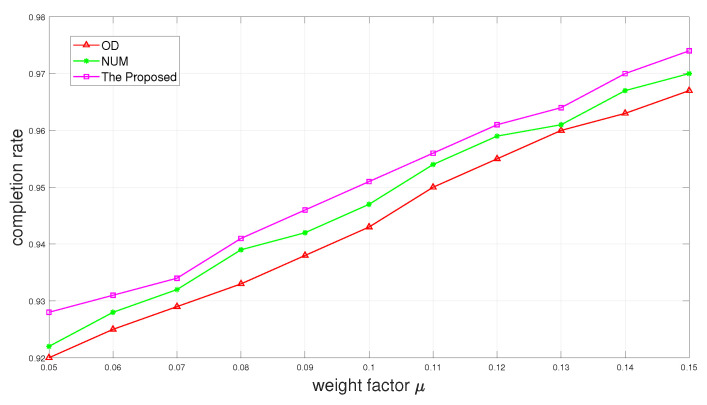
Completion rate versus weight factor μ.

**Figure 7:**
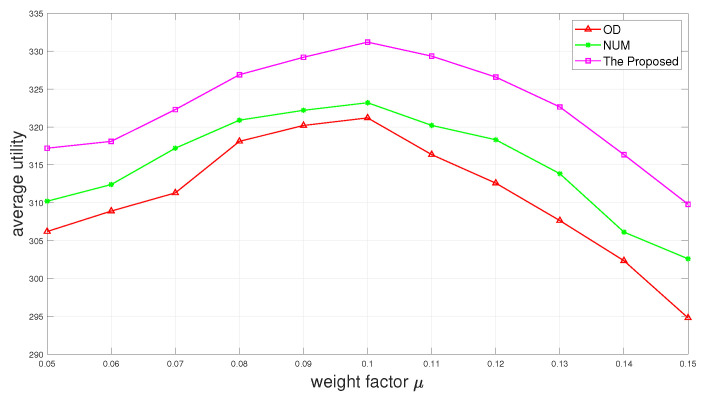
Average utility versus weight factor μ.

Figure 6 and Figure 7 show the completion rate and the average utility as a function of weight factor μ of Δt. It is depicted in Figure 6 that the completion rate promotion can be gained with the increase in the weight factor μ. Figure 7 shows that the average utility first increases and then decreases as the weight factor μ increases. It happens because when μ is too large, the time reserved for offloading also becomes larger. Although it will increase the completion rate, it also wastes part of the time, resulting in reducing overall utility.

## 8. Conclusions

This paper proposed a two-layer edge intelligence scheme to improve the efficiency of the task offloading and computing capacity allocation, and release the burden on ground devices and RSUs servers in this scenario by equipping a UAV in the air. Specifically, the first layer intelligently makes the offloading decision to tasks generated by users, and the second layer allocates the computing capacity of each PN to different tasks. Meanwhile, we proposed a weighted position optimization algorithm for the UAV movement in the system which can make the UAV perform task offloading and task processing efficiently. Simulation results demonstrated that the proposed two-layer edge intelligence scheme improved the performance. It can be seen from the simulation results that the application of UAV will be more effective when the task volume is congested. In addition, this work did not consider the collaboration between the neighbouring RSUs. Therefore, in future work, we will comprehensively consider the UAV application scenarios and energy consumption, and expand the current framework to further improve the efficiency and adaptability of vehicle task processing.

## Figures and Tables

**Table 1:** Summary of important notation.

Notation	Definition
$M_{s}$	MEC server
$U_{v}$	The V-Users
$U_{p}$	The P-User
$N_{v},N_{p}$	The set of the number of V-User or P-User
$L_{q}\left(t\right)$	The location of PN *q*
$W_{u}\left(t\right)$	The computation data size
$x_{u}^{q}\left(t\right)$	The offloading decision to the task
$w_{u}^{q}\left(t\right)$	The ratio of computing capacity allocated to the newly arrived task
$T_{u}^{o}\left(t\right)$	The offloading delay
$T_{u}^{p}\left(t\right)$	The processing delay
$T_{u}\left(t\right)$	Total service delay
Δt	Small part of time slot selected for task offloading
$C_{q}$	The computing capacity of PN *q*
$M_{q}\left(t\right)$	The maximum size of computation data can be processed in one time slot by PN *q*
$D_{q}\left(t\right)$	The remaining data size on the PN *q*
u(t)	The utility function

**Table 2:** Network parameters.

Parameter	Definition	Value
$B_{s}$	Transmission Bandwidth	10 MHz
$p_{p}$	Transmit Power of P-User	25 dBm
$p_{v}$	Transmit Power of V-User	50 dBm
$\sigma^{2}$	Noise Power	−100 dBm
$C_{v}$	Computing Capacity of V-User	0.5 GHz
$C_{p}$	Computing Capacity of P-User	0.1 GHz
$C_{r}$	Computing Capacity of RSU	2 GHz
$C_{u}$	Computing Capacity of UAV	10 GHz

## Data Availability

The data presented in this study are available upon request.
